# P2Y1 Purinergic Receptor Modulate Axon Initial Segment Initial Development

**DOI:** 10.3389/fncel.2019.00152

**Published:** 2019-04-24

**Authors:** Wei Zhang, Angela Bonadiman, María Ciorraga, María José Benitez, Juan José Garrido

**Affiliations:** ^1^Spanish National Research Council (CSIC), Department of Molecular, Cellular and Developmental Neurobiology, Instituto Cajal, Madrid, Spain; ^2^Departamento de Química Física Aplicada, Universidad Autónoma de Madrid, Madrid, Spain

**Keywords:** axon initial segment, purinergic receptors, P2Y1, ankyrinG, axon, myosin

## Abstract

Morphological and functional polarization of neurons depends on the generation and maintenance of the axon initial segment (AIS). This axonal domain maintains axonal properties but is also the place where the action potential (AP) is generated. All these functions require the AIS, a complex structure that is not fully understood. An integrated structure of voltage-gated ion channels, specific cytoskeleton architecture, as well as, scaffold proteins contributes to these functions. Among them, ankyrinG plays a crucial role to maintain ion channels and membrane proteins. However, it is still elusive how the AIS performs its complex structural and functional regulation. Recent studies reveal that AIS is dynamically regulated in molecular composition, length and location in response to neuronal activity. Some mechanisms acting on AIS plasticity have been uncovered recently, including Ca^2+^, calpain or calmodulin-mediated modulation, as well as post-translational modifications of cytoskeleton proteins and actin-associated proteins. Neurons are able to respond to different kind of physiological and pathological stimuli from development to maturity by adapting their AIS composition, position and length. This raises the question of which are the neuronal receptors that contribute to the modulation of AIS plasticity. Previous studies have shown that purinergic receptor P2X7 activation is detrimental to AIS maintenance. During initial axonal elongation, P2X7 is coordinated with P2Y1, another purinergic receptor that is essential for proper axon elongation. In this study, we focus on the role of P2Y1 receptor on AIS development and maintenance. Our results show that P2Y1 receptor activity and expression are necessary during AIS initial development, while has no role once AIS maturity is achieved. P2Y1 inhibition or suppression results in a decrease in ankyrinG, βIV-spectrin and voltage-gated sodium channels accumulation that can be rescued by actin stabilization or the modulation of actin-binding proteins at the AIS. Moreover, P2X7 or calpain inhibition also rescues ankyrinG decrease. Hence, a dynamic balance of P2Y1 and P2X7 receptors expression and function during AIS assembly and maturation may represent a fine regulatory mechanism in response to physiological or pathological extracellular purines concentration.

## Introduction

The axon initial segment (AIS) plays a crucial role in neuronal physiology, being responsible for the coordination of the whole set of inputs that a neuron receives. This unique axonal domain generates the action potential (AP; Stuart et al., 1997; Kole et al., 2008) and has a high degree of plasticity that allows the control of AP amplitude and frequency. In fact, the AIS may undergo lengthening or shortening, changes in its composition and can move away from soma to control AP response (Grubb and Burrone, 2010; Kuba et al., 2010, 2015; Del Puerto et al., 2015). Further, axonal identity and neuronal polarity depend on AIS integrity (Schafer et al., 2009). Different mental disorders (e.g., bipolar disorder, schizophrenia) and genetic diseases (e.g., Angelman syndrome) are related to AIS alterations (Kaphzan et al., 2011; van der Werf et al., 2017; Zhu et al., 2017), as well as neurodegeneration related diseases, such as stroke, brain injury or Alzheimer’s disease (Schafer et al., 2009; Sun et al., 2014; Del Puerto et al., 2015). Despite the importance of AIS, our knowledge is far from a complete understanding of its whole composition, structure and plasticity. In this sense, it remains mostly unknown which receptors and extracellular factors participate on AIS development, maintenance and modulation.

The AIS comprises around the first 20–60 microns of the axon, depending on the type of neuron and developmental stage (for a review see Leterrier, 2018). It is composed by a high density of voltage-gated sodium, potassium and calcium ion channels, and also contains among others, GABA, dopamine and serotonin receptors, which contribute to modulate APs (Rasband, 2010). These voltage-gated ion channels are anchored through interactions with AIS enriched scaffold proteins, such as AnkyrinG or PSD-93 (Garrido et al., 2003; Pan et al., 2006; Ogawa et al., 2008). AnkyrinG is the most important structural protein in the AIS and AnkyrinG suppression drives to the loss of polarity and axonal identity. Scaffold proteins also serve to anchor other membrane proteins, such as L1, neurofascin or ADAM-22 (Jenkins and Bennett, 2001; Ogawa et al., 2010). Other extracellular proteins, such as Lgi1 (Seagar et al., 2017) or brevican (Hedstrom et al., 2007) also contribute to this dense protein structure. This functional and structural membrane and submembranous scaffold are anchored to a complex cytoskeleton by βIV-spectrin (Komada and Soriano, 2002), which interacts with the actin cytoskeleton (Rasband, 2010). Although recent studies have contributed to a better knowledge of this actin cytoskeleton, whose roles in AIS maintenance, development and function are not well understood. The AIS contains regular actin rings and actin patches (Watanabe et al., 2012; Leterrier et al., 2015). Some AIS actin-interacting proteins are α-actinin-2 (Sánchez-Ponce et al., 2012), synaptopodin (Bas Orth et al., 2007) or myosins (Evans et al., 2017; Janssen et al., 2017). Also, the AIS contains a synaptopodin and actin-associated structure, the cisternal organelle (Benedeczky et al., 1994), which function is still elusive despite, changes in cisternal organelle structure occurs during development and AIS plasticity (Schlüter et al., 2017). Finally, a differential microtubules cytoskeleton supports this whole structure and contributes to axonal trafficking. This microtubules cytoskeleton contains acetylated and detyrosinated tubulin that confers a higher degree of stability to this cytoskeleton (Konishi and Setou, 2009; Tapia et al., 2010).

While our knowledge of this complex interplay between important structural and functional proteins of the AIS increases, we still have little information about the external regulatory mechanisms modulating the AIS and its structural and composition plasticity.

Recent studies have highlighted the role of some receptors at the AIS and outside the AIS on the control of AIS composition, integrity and structural plasticity. The function and plasticity of AIS seem to depend on the excitatory or inhibitory profile of hippocampal neurons, as well as the input they receive and their developmental stage (Grubb et al., 2011). However, the extracellular factors, receptors, and mechanisms controlling these adaptive modifications are unknown. This raises the question of what factors contribute to a physiological development and maintenance of the AIS. Recent results show that neurotransmitter receptors participate in AIS regulation. Dopamine, through D3R receptors, modulates T-type Ca^2+^ channels at the AIS contributing to neuronal output (Bender et al., 2010; Clarkson et al., 2017). Serotonin receptors (5-HT1A) also contribute to the control of AP threshold by modulating AIS cyclic-nucleotide-gated channels (HCN1) in the medial superior olive (Ko et al., 2016). In addition, 5-HT1A receptors modulate Na_v_1.2 voltage-gated sodium channels in the AIS of cortical neurons, decreasing the success rate of AP generation (Yin et al., 2017). Cannabinoid receptor 1 (CB1R) is necessary to achieve a proper ankyrinG clustering at the AIS in the early developmental stages of AIS (Tapia et al., 2017). Other neurotransmitters such as ATP, through the purinergic receptor P2X7, modulate AIS composition (Del Puerto et al., 2015). Purinergic receptors are a wide family of receptors expressed in neurons and glial cells and are important regulators of neuronal excitability. They can be divided into three subfamilies, adenosine, P2X and P2Y receptors, all activated by different neuronal and glial secreted purines (Del Puerto et al., 2013). They have an important role not only in physiological but also in pathological processes (Burnstock, 2017). Among them, the ionotropic P2X7 receptors are activated by high concentrations of extracellular ATP, while the metabotropic P2Y1 receptor is preferentially activated by ADP (Burnstock, 2007). P2X7 and P2Y1 purinergic receptors have an antagonistic role and are coordinated during initial axon elongation (del Puerto et al., 2012). While P2X7 has a detrimental effect on axonal elongation, P2Y1 promotes axonal elongation during the first stages of axonal growth. These antagonistic actions are regulated by P2X7-mediated calcium entry that inhibits adenylate cyclase 5 (AC5), and P2Y1-mediated Gq-PKCζ activation of AC5. This generates a balance that controls cAMP production and modulates PI3K-Akt-GSK3 pathway activity. Our previous work demonstrated that P2X7 activation is detrimental for ankyrinG and voltage-gated sodium channels accumulation in AIS of mature neurons (Del Puerto et al., 2015). However, P2Y1 role on AIS regulation has not been studied. The objective of our study was to investigate whether P2Y1 purinergic receptor participates on AIS development or modulation. Our results show that P2Y1 activity is necessary for initial AIS development and ankyrinG accumulation while having no significant role in mature neuron AIS. Decreased ankyrinG accumulation due to P2Y1 suppression can be prevented through P2X7 or calpain inhibition, revealing the crosstalk of P2Y1 and P2X7 during AIS development. Further, P2Y1 suppression alters F-actin distribution in AIS and decreases phosphorylation of myosin light chain (pMLC) and ankyrinG which can be prevented by F-actin stabilizing agent Jasplakinolide or protein phosphatase 1 and 2A inhibitor Calyculin A. Together with previous results on purinergic regulation (del Puerto et al., 2012; Del Puerto et al., 2015), our results suggest a necessary role for P2Y1 for the early development of the AIS. However, with neuronal maturation, the importance of P2Y1 decreases while P2X7 gains and increasing relevance in AIS maintenance and modulation.

## Materials and Methods

### Reagents and Plasmids

Reagents were obtained from the following manufacturers: Adenosine 5’-diphosphate sodium ADP (A2754), P2X7 antagonist, BBG (B0770), AC5 inhibitor, NKY80 (N2165) and PP1/PP2A phosphatases inhibitor calyculin A (C5552) were obtained from Sigma-Aldrich. P2Y Antagonist II (BPTU; 504187) was obtained from EMD Millipore. 2-Methylthioadenosine diphosphate trisodium salt (2-MeSADP; 1624), (N)-methanocarba-2MeSADP (MRS 2365; 2157), the calpain inhibitor MDL 28170 and jasplakinolide were obtained from Tocris. P2Y1 shRNA and scrambled shRNA plasmids have been previously published and validated (del Puerto et al., 2012).

### Animals

Animals were housed in a room at controlled temperature and relative humidity with alternating 12 h light and dark cycles and free access to food and water “*ad libitum*.” Animal care protocols used in our laboratory are in conformity with the appropriate national legislation (53/2013, BOE no. 1337) and guidelines of the Council of the European Communities (2010/63/UE). All protocols were previously approved by the CSIC bioethics committee.

### Neuronal Culture

Mouse hippocampal neurons were prepared as previously described (del Puerto et al., 2012; Del Puerto et al., 2015). Neurons were obtained from E17 mouse hippocampi, which were incubated in a 0.25% trypsin solution in Ca^2+^/Mg^2+^ free Hank’s buffered salt solution (HBSS) and dissociated using fire-polished Pasteur pipettes. The cells were plated on polylysine-coated coverslips (1 mg/ml) at a density of 5,000 cells/cm^2^ for 2 h in plating medium [minimum essential medium (MEM), 10% horse serum, 0.6% glucose and Glutamax-I]. Then coverslips were inverted and transferred to culture dishes containing astrocytes. Astrocytes medium was replaced by neuronal culture medium 24 h before neuronal culture (Neurobasal medium, B27 supplement, Glutamax-I). To avoid contact between neurons and astrocytes, paraffin beads were placed on coverslips before neuronal plating. Five micromolar 1-β-D-arabinofuranosylcytosine (AraC) was added after 2 days in culture to avoid glial cells proliferation. One-third of neuronal medium was replaced every week. Pharmacological treatments were applied as described in the “Results” section. In the case of pharmacological treatments in the absence of glial cell layer, coverslips were transferred to plates containing glial cells-conditioned medium. Primary hippocampal neurons were nucleofected using the Amaxa nucleofector kit for primary mammalian neural cells (Amaxa Bioscience) according to the manufacturer’s instructions. Nucleofection was performed using 3 μg of total DNA and 3 × 10^6^ cells for each nucleofection. Neurons were plated at a density of 10,000 cells/cm^2^ as described above. Nucleofection efficiency was ~15% of neurons, based on the number of GFP-positive neurons. For lipofection, neurons were plated at a density of 20,000 cells/cm^2^ and transfected at 7 or 10 DIV. Lipofection was performed using Lipofectamine 2000 (Life Technologies) and 3 μg of total DNA following manufacturer instructions.

### Immunofluorescence

Neurons from each experiment, containing all experimental conditions to be compared, were fixed in 4% PFA. To standardize staining, all these coverslips were treated at the same time for immunofluorescence following the same conditions. Briefly, coverslips were treated for 10 min with 50 mM NH_4_Cl and incubated in blocking buffer (0.22% gelatine, 0.1% Triton X-100 in PBS) for 30 min, before incubation with primary antibodies for 1 h at room temperature in blocking buffer. The primary antibodies used were: chicken anti-MAP2 (1:10,000, Abcam, ab5392), mouse anti-ankyrinG (1:100) from NeuroMab (clone N106/36), mouse anti-pan sodium channel (1:100) from Sigma (clone K58/35), rabbit anti-pMLC (1:200) from Thermo Fisher (PA5-17727) and rabbit βIV-spectrin (1:1,000; a gift from Dr. M. Rasband, Baylor College of Medicine, Houston, TX, USA). The secondary antibodies used were a goat or donkey anti-mouse, anti-rabbit or anti-chicken Alexa-Fluor 488, 594, or 647 (1:1,000). Phalloidin Alexa-Fluor 594 was used at a concentration of 1:100. Images from each immunofluorescence were acquired on a Leica SP5 confocal microscope maintaining the same acquisition parameters to compare intensities. Figures were prepared for presentation using the Adobe CS4 software.

### Dendrites and AIS Measurements

Quantification of fluorescence intensity at the AIS was performed on confocal images of neurons from at least three independent experiments. Neurons were randomly acquired without any bias by scanning each coverslip from top to bottom. All confocal images within the same experiment and immunofluorescence were acquired using exactly the same parameters of laser intensity, excitation and emission detection during the same working session for each Alexa fluorophore. Parameters were set in control neurons and maintained for the other experimental groups. After completing images acquisition of all conditions, ankyrinG and other AIS proteins staining were quantified using ImageJ software. We drew a line starting at the limit of neuronal soma identified by MAP2 staining and extended it along the ankyrinG staining or the GFP signal of the axon. Data from every 0.16 μm along the first 40 μm were obtained and smoothed using the Sigmaplot software to obtain average ankyrinG fluorescence intensity every 1 μm. Data were normalized in each neuron, within the same immunofluorescence, considering the value of maximum mean fluorescence in control neurons to be 100%. Total fluorescence intensity for each neuron was obtained by adding ankyrinG fluorescence values from 0 to 40 μm. In some experiments, the total ankyrinG intensity was calculated within each AIS length and data normalized to AIS length of each neuron. AIS start, end and maximum fluorescence intensity were determined following the criteria described in Grubb and Burrone (2010). Taking 100% fluorescence as the maximum fluorescence intensity point, start and end points were defined as the points were fluorescence intensity is lower than 33%. For actin patches quantification, neurons were stained with Phalloidin-Alexa 594 (1:100) and ankyrinG to detect the AIS. Puncta of relatively uniform diameter of less than 3 μm within AIS were considered to be patches (Balasanyan et al., 2017). To avoid any possible loss of actin patches by lower F-actin intensity, image intensity was increased for the purpose of identification and quantification of all possible actin patches.

Dendrites lengths were obtained based on MAP2 staining using NeuronJ software to measure the length of the dendritic arbor in each neuron. All dendrites and ramifications in each neuron were traced using NeuronJ and their total length added. GFP signal of neurons under analysis allowed discriminating from crossing dendrites from other neurons. In order to study correlations between dendrites and AIS fluorescence, both data were obtained from the same neurons.

### Statistical Analysis

All statistical analyses were carried out in Sigmaplot v12.5 (Systat Software Inc.) and Prism 5 (GraphPad Software, Inc., La Jolla, CA, USA). Data for each independent sample were obtained from at least three independent experiments. For pharmacological experiments, data from each experiment were collected from at least 30 cells (between 30 and 50 cells) in each experimental condition. For nucleofection or lipofection experiments around 25 neurons from each experiment in each experimental condition were analyzed. Statistical analysis was performed by *t*-test for two group comparisons and one-way ANOVA for multiple group comparisons. When data were non-normally distributed, non-parametric tests were used: Mann-Whitney Rank test for two independent samples and Kruskal-Wallis for analysis of multiple groups. In the analysis of multiple comparisons, a *post hoc* analysis was performed using Dunn’s test. All *p-values* were adjusted to account for multiple comparisons. Cell-to-cell analysis of dendrite length and ankyrinG fluorescence was performed using Prism 5 and Sigmaplot v12.5. First, we tested the normality of data distribution on each variable using a Shapiro-Wilk or Kolmogorov-Smirnov normality test. As not all data passed normality test, the correlation was analyzed using the Pearson correlation function or Spearman correlation function when data failed normality test. Differences were considered significant when *p* < 0.05.

## Results

### ADP Activation of P2Y1 Increases AnkyrinG Levels in the Developing Axon Initial Segments

**Figure 1:**
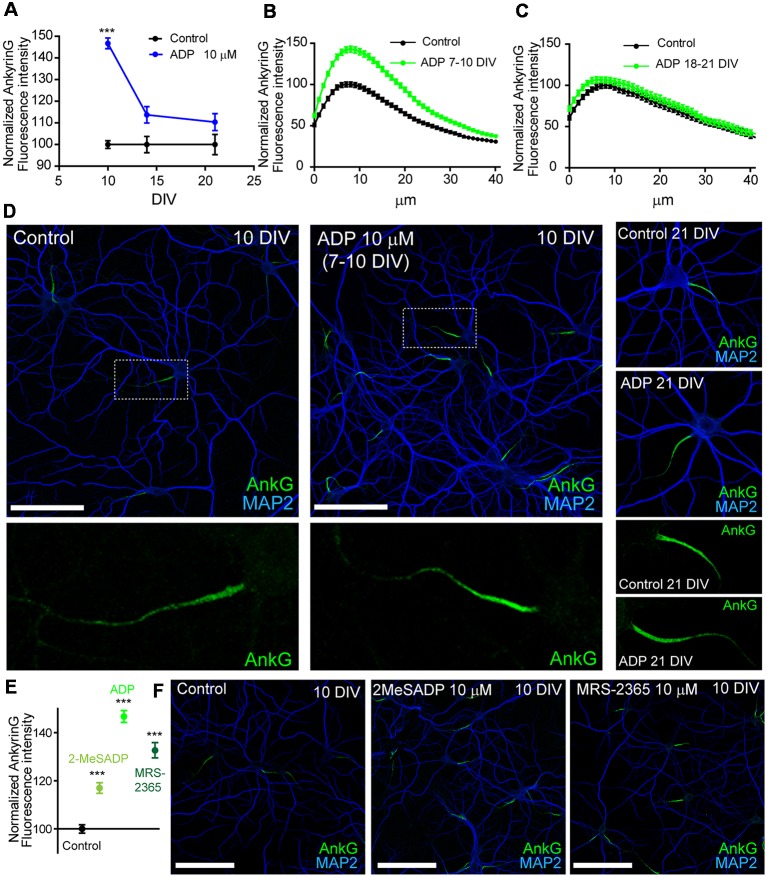
ADP and P2Y1 agonists potentiates ankyrinG expression during early axon initial segment (AIS) development. **(A)** Normalized ankyrinG fluorescence intensity in 10, 14 and 21 DIV hippocampal neurons. Neurons were treated with ADP for 3 days before fixation (blue symbols). Data were acquired from three independent experiments (30 neurons/experimental condition in each experiment). Same pool of neurons was used for each experiment and fixed at different times. All images were acquired by confocal microscopy using the same fluorescence parameters. Statistical differences were analyzed by a Kruskal-Wallis test followed by a Dunn’s multiple comparisons post-test. Adjusted *p* values: ***p < 0.001. **(B,C)** Normalized AnkyrinG intensity profile along the AIS of 10 DIV **(B)** and 21 DIV **(C)** hippocampal neurons in the presence (green line) or absence (black line) of 10 μM ADP treatments. **(D)** Control and ADP treated 10 DIV and 21 DIV neurons stained with MAP2 (blue) and ankyrinG antibodies (green). Scale bar = 100 μm. Four times-magnification of the ankyrinG staining (green) at the AIS is shown below images. **(E)** Normalized ankyrinG intensity at the AIS of 10 DIV neurons treated with ADP and P2Y1 agonists 2-methylthioadenosine diphosphate trisodium salt (2MeSADP) or MRS-2365 from 7 to 10 DIV. Data were acquired from three independent experiments (30 neurons/experimental condition in each experiment). ***p < 0.001, two-tail *t*-test. **(F)** 10 DIV hippocampal neurons stained with MAP2 (blue) and ankyrinG antibodies (green) treated with 2MeSADP (10 μM) or MRS-2365 (10 μM). Data in graphs are represented as the mean ± SEM.

A previous study demonstrated that the AIS of mature cultured hippocampal neurons (21 DIV) is regulated by ATP and P2X7 receptor (Del Puerto et al., 2015). Due to the coordinated action of ATP and ADP during initial axon elongation, we have investigated whether ADP and P2Y1 play a role in AIS regulation. Hippocampal neurons were treated with vehicle or ADP 10 μM during the 3 days prior fixation at 10, 14 or 21 DIV. AnkyrinG levels were then analyzed after immunofluorescence (Figures 1A–D). ADP treatment increased ankyrinG fluorescence intensity at the AIS in neurons treated from 7 to 10 DIV (146.74 ± 2.48%) compared to 10 DIV control neurons (100 ± 1.76%). However, ADP treatment in 14 DIV neurons had no significant effect in ankyrinG levels (113.74 ± 3.81%) vs. 14 DIV control neurons (100 ± 3.78%), as also happened for ADP treatment in 21 DIV neurons (110.38 ± 3.89% vs. AIS of 10 and 21 DIV neurons (Figures 1B,C). Next, we treated neurons with two more P2Y1 agonists, 2MeSADP (10 μM) and MRS-2365 (10 μM). Both agonists also increased significantly ankyrinG intensity at the AIS of 7–10 DIV treated neurons (Figures 1E,F), suggesting a P2Y1 mediated effect of ADP.

### P2Y1 Receptor Suppression or Inhibition Decreases AnkyrinG Accumulation at the AIS During Initial Developmental Stages

**Figure 2:**
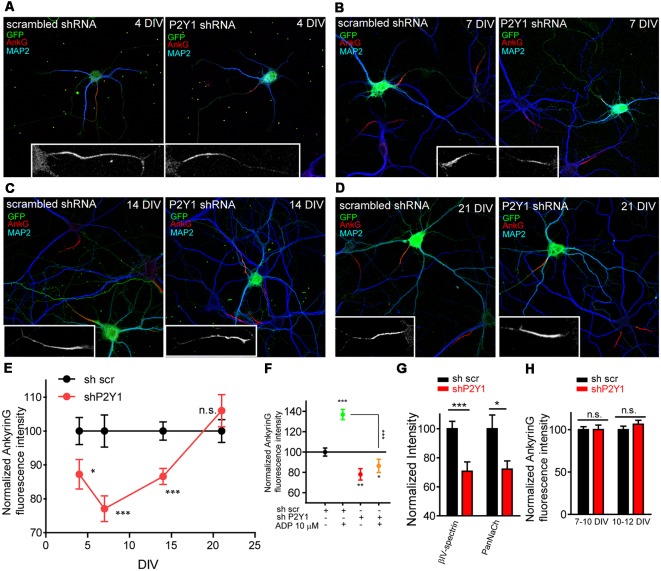
P2Y1 receptor is necessary to maintain ankyrinG density during early stages of AIS development. **(A–D)** 4, 7, 14 and 21 DIV hippocampal neurons nucleofected with scrambled and P2Y1 shRNAs. After nucleofection neurons were kept in culture for 4 **(A)**, 7 **(B)**, 14 **(C)** and 21 days **(D)** and stained with MAP2 (blue) and ankyrinG (red) antibodies. Nucleofected neurons were identified by GFP expression (green). Inserts show magnifications of AISs (gray) of each neuron. **(E)** Normalized ankyrinG intensity at different days post-nucleofection with scrambled shRNA (shscr, black) or P2Y1 shRNA (shP2Y1, red). shP2Y1 data are normalized to their respective shscr controls at each developmental stage. *p < 0.05, **p < 0.01, ***p < 0.001, two-tail *t*-test. **(F)** Normalized ankyrinG intensity in 10 DIV neurons expressing shscr or shP2Y1 plasmids, treated with vehicle or ADP from 7 to 10 DIV. *p < 0.05, **p < 0.01, ***p < 0.001, two-tail *t*-test. Data were acquired from three independent experiments (30 neurons/experimental condition in each experiment). **(G)** βIV-spectrin and voltage-gated sodium channels (PanNaCh) normalized intensity in 7 DIV control shscr and shP2Y1 expressing neurons. *p < 0.05, ***p < 0.001, two-tail *t*-test. Data were acquired from three independent experiments (at least 25 neurons/experimental condition in each experiment). **(H)** Normalized ankyrinG intensity in 10 and 12 DIV hippocampal transfected at 7 or 10 DIV, respectively, to express shscr or shP2Y1 interference RNAs. Data were acquired from three independent experiments (at least 20 neurons/experimental condition in each experiment). n.s., not significant, two tail *t*-test. Data in graphs are represented as the mean ± SEM.

In order to ensure that ADP effect was due to P2Y1 receptor activation, we nucleofected neurons before plating using scrambled (shscr) or P2Y1 (shP2Y1) interference RNAs that also express GFP. Neurons were left in culture for 4, 7, 14 or 21 DIV, fixed and stained with MAP2 and ankyrinG antibodies (Figures 2A–D). P2Y1 interference RNA significantly decreased ankyrinG intensity between 4 and 14 DIV (Figure 2E), with the maximum reduction found at 7 DIV (77.07 ± 3.79%) compared to 100 ± 4.75% in sh scrambled 7 DIV control neurons. Fourteen DIV neurons recovered when compared to 7 DIV neurons (86.58 ± 2.36%), and no significant change was observed in 21 DIV neurons. To ascertain that ADP treatment has no effect on ankyrinG intensity after P2Y1 suppression, nucleofected 7 DIV neurons were treated with ADP 10 μM for 3 days. Neurons nucleofected with scramble shRNA increased ankyrinG intensity after ADP treatment, while those expressing shP2Y1 did not respond to ADP treatment (Figure 2F). Next, we checked whether the fluorescence intensity of ankyrinG interacting proteins, βIV-spectrin and voltage-gated sodium channels, was also reduced in 7 DIV neurons (Figure 2G). Both proteins significantly decreased their expression at the AIS after P2Y1 suppression (72.19 ± 5.67%, panNaCh and 70.69 ± 6.46%, βIV-spectrin) and the percentage was similar to ankyrinG (Figure 2E). To rule out the possibility that shP2Y1 efficiency was progressively lost during *in vitro* development, we introduced control scrambled shRNA and P2Y1 shRNA by lipofection in 7 or 10 DIV neurons. Neurons were left 2–3 days and ankyrinG signal was analyzed (Figure 2H). Lipofected neurons expressing shP2Y1 did not show any reduction of ankyrinG levels at neither 10 or 12 DIV, confirming that in cultured hippocampal neurons P2Y1 exerts its action only during the initial steps of AIS development. This timing correlates with the stages prior to which the AIS diffusion barrier is developed at 10 DIV (Brachet et al., 2010).

**Figure 3:**
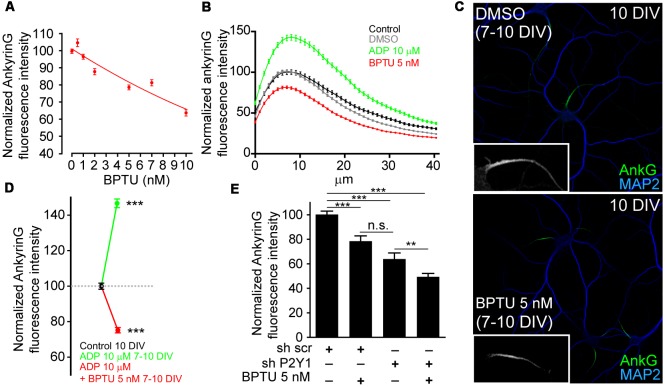
P2Y1 pharmacological inhibition decreases ankyrinG accumulation at the AIS. **(A)** Normalized ankyrinG intensity on 10 DIV hippocampal neurons treated from 7 DIV with increasing concentrations of the P2Y1 antagonist, BPTU (0.5, 1, 2, 5, 7 and 10 nM). Concentrations higher than 10 nM affected the morphology of supporting glial cells and were discarded to avoid secondary effects on neurons. **(B)** AnkyrinG intensity profile along the AIS of hippocampal neurons treated from 7 to 10 DIV with vehicle black line), DMSO (BPTU vehicle, gray line), ADP (green line) and BPTU (red line). **(C)** 10 DIV neurons stained with MAP2 (blue) and ankyrinG antibodies (green) and treated with vehicle (control) or 5 nM BPTU from 7 to 10 DIV. Inserts show a magnification of the respective AIS. **(D)** Normalized ankyrinG intensity on 10 DIV hippocampal neurons treated from 7 DIV with 10 μM ADP and BPTU 5 nM, alone or in combination. **(E)** Normalized ankyrinG intensity on 10 DIV shscr or shP2Y1 nucleofected hippocampal neurons treated with BPTU from 7 DIV. Data were acquired from three independent experiments (at least 30 neurons/experimental condition in each experiment). **p < 0.01, ***p < 0.001, n.s. not significant, two-tail *t*-test. Data in graphs are represented as the mean ± SEM.

In view of these results, we next checked whether P2Y1 pharmacological inhibition was enough to reduce ankyrinG levels. For this purpose, we used the non-nucleotide allosteric and none ADP-competitive P2Y1 antagonist, BPTU (1-(2-(2-(tert-butyl)phenoxy)pyridin-3-yl)-3-(4-(trifluoromethoxy)phenyl)urea; Mañé et al., 2016). First, we determined the most efficient dose in cultured hippocampal neurons. Seven DIV neurons were treated for 3 days with BPTU at concentrations of 0.5, 1, 2, 5, 7, 10 and 20 nM and ankyrinG levels were evaluated (Figure 3A). AnkyrinG levels decreased linearly with increasing BPTU concentration. BPTU doses under 10 nM did not affect neuronal viability, but concentrations higher than 10 nM had a detrimental effect on neuronal survival due to alterations on glial cell layer morphology and viability (data not shown). Consequently, we decided to inhibit P2Y1 using 5 nM BPTU treatments in 7 DIV neurons for 3 days (Figure 3C). While 7–10 DIV neurons increased ankyrinG levels after ADP treatment, 5 nM BPTU reduced ankyrinG levels (Figure 3B) to similar levels (75.21 ± 1.54%) than those observed in shP2Y1 neurons (77.07 ± 3.79%, Figure 2E, or 63.86 ± 4.95%, Figure 3E). No difference was observed between the ADP and BPTU vehicles, water and DMSO, respectively (Figure 3B). Moreover, BPTU treatment impaired the positive effect of ADP on ankyrinG (Figure 3D). Finally, we combined shP2Y1 with BPTU and again assessed ankyrinG. 10 DIV neurons expressing shP2Y1 had decreased ankyrinG expression (63.86 ± 4.95%), which was significantly reduced (49.38 ± 2.76%) by BPTU treatment in shP2Y1 neurons (Figure 3E). The fact that BPTU has an additive effect with P2Y1 suppression may be explained by the fact that still some P2Y1 receptors are available and BPTU is a potent P2Y1 inhibitor. shP2Y1 has an efficiency of 70% reducing P2Y1 expression in cell lines (del Puerto et al., 2012). On the other hand, it is the first time that BPTU is used in neurons. We can not discard that BPTU is partially affecting other purinergic receptors. However, BPTU impairs ADP-mediated increase of ankyrinG fluorescence. Therefore, our results suggest that P2Y1 activation is necessary to maintain ankyrinG levels at the AIS during the first stages of AIS development. This led to the question which are the mechanisms or developmental events that are under P2Y1 regulation that contribute to AIS proteins accumulation.

### AnkyrinG and Dendrites or AIS Length do Not Correlate in Neurons Lacking P2Y1 Receptor

**Figure 4:**
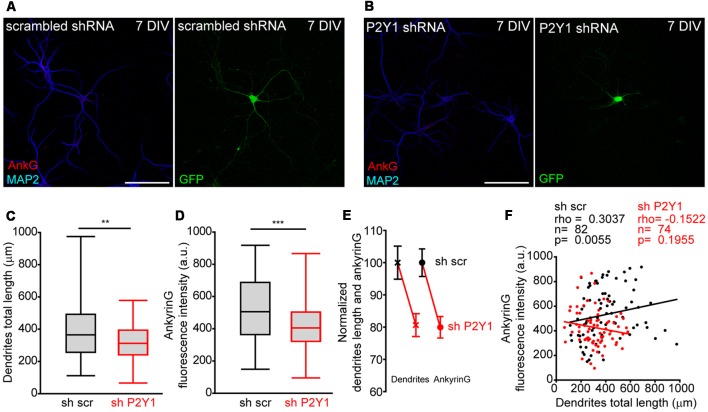
No correlation between P2Y1 suppression-mediated decrease of dendrites length and ankyrinG density. **(A,B)** Representative images of 7 DIV hippocampal neurons nucleofected with scrambled or P2Y1 shRNAs. After nucleofection neurons were kept in culture for 7 DIV and stained with MAP2 (blue) and ankyrinG (red) antibodies. Nucleofected neurons were identified by GFP expression (green). **(C,D)** Box-plots represent total dendritic length **(C)** and ankyrinG intensity **(D)** of 7 DIV scrambled sh (sh scr) or shP2Y1 nuclefected neurons from the same pools in three different experiments. Line inside the box represents the median, and upper and bottom lines represent the maximum and minimum values. **p < 0.01, ***p < 0.001, two-tail *t*-test. **(E)** Normalized mean ± SEM of dendrites length and ankyrinG intensity of data represented in **(C,D)**. **(F)** Correlation graph of dendrites length and ankyrinG intensity for each individual neuron analyzed in the study. Control neurons (shscr) are represented in black and neurons expressing P2Y1 interference RNA (shP2Y1) are in red. Data were analyzed first with two normality tests (Shapiro-Wilk and Kolmogorov-Smirnov). Not all data sets did fit a normal distribution and a non-parametric correlation test (Spearman) was performed. Regression lines are shown for shscr (black) and shP2Y1 (red) neurons. The number of neurons, Spearman *r* coefficient and *p* values are indicated in **(F)**. A positive correlation is observed in control neurons, while there is no significant correlation in neurons expressing shP2Y1 interference RNA.

P2Y1 receptor is not detected at the AIS but is located in dendrites, distal axon and presynaptic terminals in cultured hippocampal neurons (del Puerto et al., 2012). We recently demonstrated that ankyrinG decrease at AIS was correlated with impaired dendrite development mediated by CB1R inhibition (Tapia et al., 2017) and previous studies demonstrated that P2Y1 modulate neuronal morphology during the initial stages of development (del Puerto et al., 2012). Dendrites start their development in cultured hippocampal neurons at 4 DIV (Dotti et al., 1988). In order to test whether P2Y1 may control dendrite development and also influence AIS development, we measured the cell-to-cell dendritic length and ankyrinG fluorescence intensity in 7 DIV shscr and shP2Y1 nucleofected neurons (Figures 4A,B). As happened when CB1 expression was reduced (Tapia et al., 2017), dendrites of 7 DIV shP2Y1 neurons were shorter (320.2 ± 14.1 μm) than in control neurons (397.1 ± 20.37 μm, Figures 4C,E). Moreover, ankyrinG expression at the AIS was also reduced by 20% (Figures 4D,E). Even though a similar percentage reduction of dendrites length and ankyrinG was observed (Figure 4E), we analyzed both parameters in each individual neuron (Figure 4F). While we found a significant correlation between dendritic arbor length and ankyrinG expression in sh scrambled control neurons (Spearman *r* = 0.3037, *p* = 0.0055), there was no significant correlation in shP2Y1 neurons (Spearman *r* = −0.1522, *p* = 0.1955).

**Figure 5:**
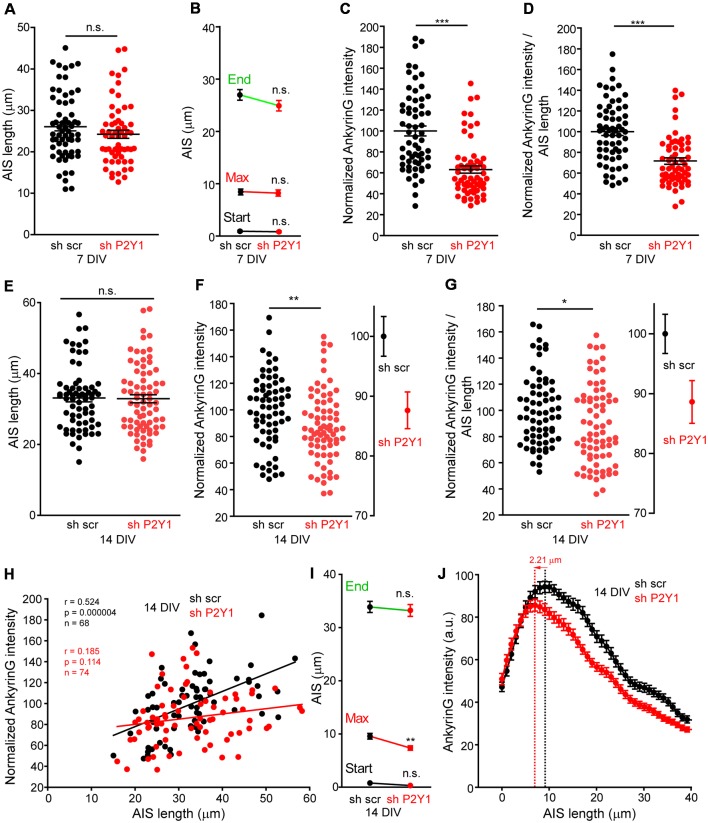
No correlation between P2Y1 suppression-mediated decrease of ankyrinG density and AIS length. **(A)** Graph represents AIS length of each 7 DIV neuron nucleofected with scrambled sh (black dots) or shP2Y1 (red dots). Lines represent the mean ± SEM. **(B)** Graph represents the mean ± SEM of AIS start point, maximum intensity point and end point of AIS in 7 DIV scrambled shRNA or P2Y1 shRNA nucleofected neurons. **(C)** Normalized ankyrinG intensity within the respective AIS length of neurons represented in **(A)**. **(D)** Normalized AnkyrinG/AIS length ratio for each 7 DIV scrambled shRNA or P2Y1 shRNA nucleofected neuron. **(E)** Graph represents AIS length of each 14 DIV neuron nucleofected with scrambled sh (black dots) or shP2Y1 (red dots). Lines represent the mean ± SEM. **(F)** Normalized ankyrinG intensity within the respective AIS length of neurons represented in **(E)**. Adjacent graph represents the mean ± SEM. **(G)** Normalized AnkyrinG/AIS length ratio for each 14 DIV scrambled shRNA or P2Y1 shRNA nucleofected neuron. Adjacent graph represents the mean ± SEM. **(H)** Correlation graph of AIS length and ankyrinG intensity for each individual 14 DIV neuron nucleofected with scrambled (black dots) or P2Y1 (red dots) shRNAs. Values are the same represented in **(E,F)**. Data were analyzed first with two normality tests (Shapiro-Wilk and Kolmogorov-Smirnov). All data sets did fit a normal distribution and a Pearson correlation test was used. Regression lines are shown for shscr (black) and shP2Y1 (red) neurons. The number of neurons, Pearson *r* coefficient and *p* values are indicated in **(H)**. A positive correlation is observed in control neurons, while there is no significant correlation in neurons expressing shP2Y1 interference RNA. **(I)** Graph represents the mean ± SEM of AIS start point, maximum intensity point and end point of AIS in 14 DIV scrambled shRNA or P2Y1 shRNA nucleofected neurons. Note a significant proximal shift of the maximum intensity point. **(J)** AnkyrinG profile along AIS length of 14 DIV neurons, indicating the proximal shift of the maximum ankyrinG intensity. Data are represented as the mean ± SEM of ankyrinG intensity every 1 micron. *p < 0.05, **p < 0.01, ***p < 0.001, n.s., non-significant.

On the other hand, the AIS is known to respond to stimulus or absence of stimulus with plasticity mechanisms that include changes in composition, length and position (Grubb and Burrone, 2010; Kuba et al., 2010; Del Puerto et al., 2015). Therefore, we analyzed AIS length in 7 and 14 DIV shscr and shP2Y1 neurons (Figure 5). No significant AIS length differences were found in 7 DIV shP2Y1 neurons (26.06 ± 1.03 μm) compared to control 7 DIV sh scrambled neurons (23.52 ± 0.97 μm), neither in AIS position or maximum ankyrinG intensity position (Figures 5A,B). No significant change in AIS length was also obtained in 14 DIV neurons, being AIS length around 33 μm in sh scrambled and shP2Y1 neurons (Figure 5E). However, in 14 DIV neurons, P2Y1 suppression generated a significant proximal shift of ankyrinG maximum intensity of 2–3 μm (7.37 ± 0.41 μm vs. 9.58 ± 0.53 μm in sh scrambled neurons, Figure 5I), which can be observed in the mean profile of ankyrinG expression along the AIS (Figure 5J). Despite no AIS length mean changes were observed between scrambled sh and shP2Y1 neurons, there is variability of AIS length within each experimental group. Thus, we analyzed ankyrinG intensity within the AIS length of each neuron (Figures 5C,F). Our results show a significant reduction of the mean ratio ankyrinG/AIS length in shP2Y1 neurons at 7 DIV (around 30%, Figure 5D) and 14 DIV (around 10%, Figure 5G). Furthermore, we analyzed whether AIS length and ankyrinG intensity are correlated in 14 DIV scrambled sh and shP2Y1 neurons (Figure 5H). Both parameters were significantly correlated in control scrambled sh neurons, while no significant correlation was found in shP2Y1 neurons, demonstrating that ankyrinG intensity reduction in shP2Y1 neurons is not related to changes in AIS length. This data suggest that besides neuronal morphology regulation, P2Y1 receptor maintain other mechanisms to regulate AIS development.

### P2X7 Inhibition Recovers AnkyrinG Expression

**Figure 6:**
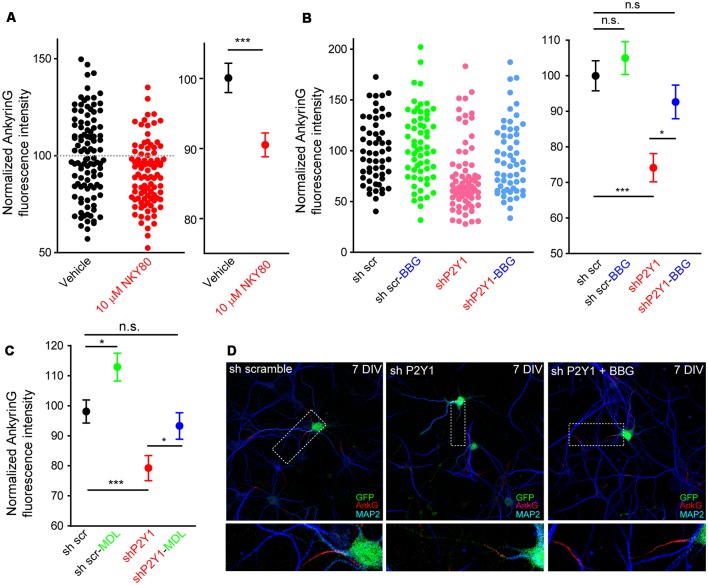
P2X7 inhibition recovers ankyrinG density in shP2Y1 nucleofected neurons. **(A)** Scatter plot showing normalized ankyrinG intensity in 10 DIV control neurons or treated with the adenylate cyclase 5 (AC5) inhibitor NKY80 (10 μM) from 7 to 10 DIV. Mean ± SEM for each condition is shown in the adjacent graph. ***p < 0.001, n.s. no significant, two-tail *t*-test. **(B)** Scatter plot of normalized ankyrinG intensity in 7 DIV transfected with scrambled shRNA or P2Y1 shRNA and treated with the P2X7 antagonist, BBG (100 nM) from 6 to 7 DIV. Mean ± SEM for each condition is shown in the adjacent graph. Statistical differences were analyzed by a Kruskal-Wallis test followed by a Dunn’s multiple comparisons post-test. *p < 0.05, ***p < 0.001, n.s., non-significant. **(C)** Mean ± SEM of normalized ankyrinG intensity in 7 DIV nucleofected with scrambled shRNA or P2Y1 shRNA and treated with the calpain inhibitor, MDL-28170 (50 nM) from 6 to 7 DIV. Statistical differences were analyzed by a Kruskal-Wallis test followed by a Dunn’s multiple comparisons post-test. *p < 0.05, ***p < 0.001, n.s., non-significant. **(D)** Representative images of scrambled shRNA or P2Y1 shRNA treated with the P2X7 antagonist, BBG (100 nM). Neurons were stained with MAP2 (blue) and ankyrinG (red) antibodies. Nucleofected neurons were identified by GFP signal (green). Axon initials segments were magnified and presented in panels below.

Our previous results showed that P2Y1 suppression produced a shorter axon, which was recovered by P2X7 inhibition with BBG (del Puerto et al., 2012). P2Y1-Gq and P2X7-mediated calcium entry are coordinated through AC5 activation (P2Y1) or inhibition (P2X7). This generates a cAMP balance that modulates the PI3K/Akt/GSK3 pathway. In addition, P2X7 modulate ankyrinG expression at the AIS through a calcium-calpain-dependent mechanism (Del Puerto et al., 2015). In order to test whether the same coordinated pathway is also related to AIS modulation, we treated neurons from 7 to 10 DIV with the adenylate cyclase inhibitor NKY80 (10 μM) and analyzed ankyrinG (Figure 6A). NKY80 significantly reduced ankyrinG expression by around 10%, suggesting a coordinated action of P2Y1 and P2X7. Next, 6 DIV sh scrambled and shP2Y1 neurons were treated for 24 h with BBG (100 nM), a well-characterized P2X7 inhibitor (Figures 6B,D). P2X7 inhibition resulted in a non-significant trend to increase ankyrinG fluorescence in sh scrambled neurons (105.0 ± 4.59%), while P2Y1 suppression (shP2Y1 neurons) reduced ankyrinG fluoresecence around 25% (74.13 ± 3.98%). Interestingly, 24 h inhibition of P2X7 recovered ankyrinG intensity to control levels (92.7 ± 4.71%, Figures 6B,D). Further, 24 h treatment with the calpain inhibitor MDL-28170 (50 nM) recovered ankyrinG fluorescence (95.1 ± 4.49%) in shP2Y1 neurons (Figure 6C). This data suggests a coordinated and antagonistic action of P2Y1 and P2X7 receptors during initial stages of AIS development, which may depend on the amount of ATP and ADP in extracellular medium and their expression levels during AIS development. However, P2X7 inhibition did not completely recover ankyrinG intensity as previously observed in mature neurons (Del Puerto et al., 2015), suggesting that P2X7 participation is limited in young neurons. Thus, other AIS intrinsic mechanisms may be regulated by P2Y1 in AIS initial developmental stages.

### P2Y1 Suppression Modifies AIS Actin Cytoskeleton and Myosin II Light Chain Phosphorylation

**Figure 7:**
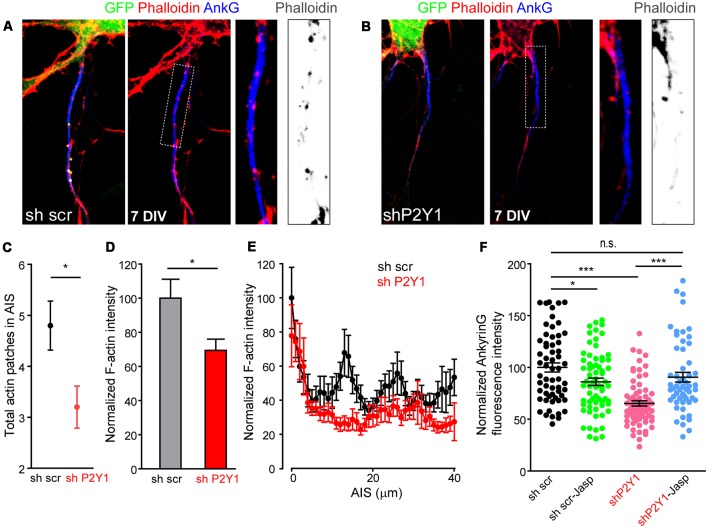
P2Y1 suppression alters AIS F-actin. **(A,B)** Representative images of 7 DIV neurons nucleofected with scrambled **(A)** or P2Y1 **(B)** shRNAs. AISs were identified by ankyrinG staining (blue) and Phalloidin-Alexa594 served to identify F-actin (red). Nucleofected neurons were identified by their GFP expression (green). Panels show AIS region of the respective neurons and a 2.5× magnification of AIS. Gray scale images show phalloidin staining at the respective AISs. **(C)** Graph represents the mean ± SEM number of actin patches identified in scrambled (black, *n* = 20) and shP2Y1 (red, *n* = 20) AISs. *p < 0.05, two-tail *t*-test. **(D)** Total AIS F-actin intensity based on Phalloidin-Alexa 594 fluorescence intensity along ankyrinG signal in 7 DIV shscr and shP2Y1 neurons. *p < 0.05, two-tail *t*-test. **(E)** Plot profile of F-actin intensity along AIS in 7 DIV scrambled shRNA (*n* = 24, black) or P2Y1 shRNA (*n* = 22, red) neurons. Data are represented as the mean ± SEM of values every micron. **(F)** Scatter plot and mean ± SEM representing normalized ankyrinG intensity of each 7 DIV scrambled shRNA (sh scr) or P2Y1 shRNA (shP2Y1) nucleofected neuron, treated or not with the actin stabilizer jasplakinolide (10 nM) during 4 h prior to fixation. Statistical differences were analyzed by a Kruskal-Wallis test followed by a Dunn’s multiple comparisons post-test. *p < 0.05, ***p < 0.001, n.s., non-significant.

Recently, actin dynamics at the AIS were related to AIS plasticity (Berger et al., 2018). Activity-dependent AIS plasticity can produce a dramatic reorganization of the actin periodic structure (Berger et al., 2018) and P2Y1 signaling pathways can activate the RhoGTPase Rac, which modulates actin dynamics in migration and differentiation processes (Soulet et al., 2005). To understand whether our results can be attributable to actin cytoskeleton modifications mediated by P2Y1 receptor, we stained neurons 7 DIV scrambled sh and shP2Y1 neurons with fluorescence phalloidin (Figures 7A,B) and quantified F-actin intensity along the AIS, as well as the number of actin patches (Figures 7C,D). Total F-actin intensity was reduced by around 30% in shP2Y1 neurons (Figure 7D), and so was ankyrinG intensity (Figure 7F). Besides, the F-actin intensity decrease in the AIS was confirmed by the profile (Figure 7E). While actin patches were visible in control AISs, these were highly reduced in intensity and visibility in shP2Y1 neurons (Figures 7A,B). Actin patches quantification, independently of their intensity, showed that scrambled sh neurons contained 4.8 ± 0.48 actin patches, while shP2Y1 neurons only 3.2 ± 0.41 patches (Figure 7C). If loss of P2Y1-dependent stabilization of the actin cytoskeleton results in the decrease in ankyrinG, then it may be recovered by actin stabilization. Thus, we treated scrambled and shP2Y1 neurons with an F-actin stabilizing agent, Jasplakinolide (10 nM) for 4 h (Figure 7F). AnkyrinG expression in shP2Y1 neurons was significantly recovered by Jasplakinolide treatment (90.6 ± 4.7%) when compared to shP2Y1 neurons with no treatment (65.27 ± 2.59%, *p* = 0.0002). After recovery, the level was equivalent to sh scrambled control neurons (100 ± 4.36%). In control sh scrambled neurons, Jasplakinolide generated a decrease in ankyrinG expression (around 14%), which may be due to a deregulation of actin dynamics and higher actin stabilization.

**Figure 8:**
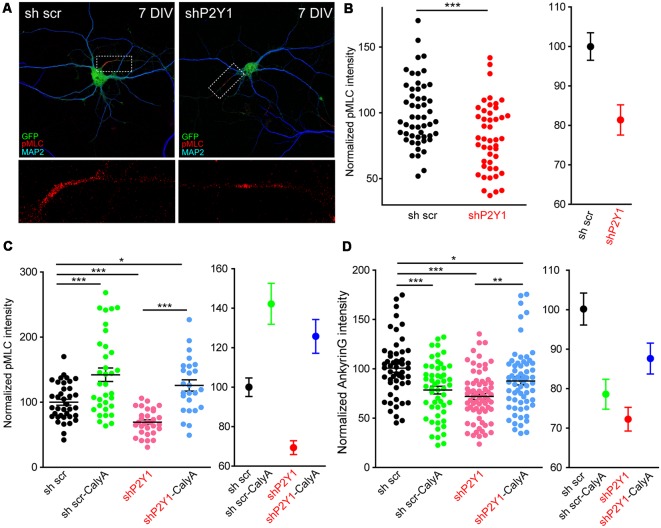
P2Y1 is necessary to maintain myosin light chain (MLC) phosphorylation at the AIS. **(A)** Representative images of 7 DIV scrambled shRNA or P2Y1 shRNA nucleofected neurons showing phosphorylation of MLC (pMLC) staining in the AIS. Neurons were stained with MAP2 (blue) and pMLC (red) antibodies. Nucleofected neurons were identified by GFP signal (green). pMLC staining at axon initials segments was magnified and presented in panels below (red). **(B)** Scatter plot for each neuron (left) and mean ± SEM (right) representing normalized pMLC intensity at the AIS of 7 DIV neurons nucleofected with scrambled shRNA or P2Y1 shRNA. ***p < 0.001, two-tail *t*-test. **(C)** Scatter plot representing normalized pMLC intensity at the AIS of 7 DIV neurons nucleofected with scrambled shRNA or P2Y1 shRNA and treated with 1 nM calyculinA (CalyA) for 3 h before fixation. Mean ± SEM of these values is represented in the adjacent graph. **(D)** Scatter plot representing normalized ankyrinG intensity at the AIS of 7 DIV neurons nucleofected with scrambled shRNA or P2Y1 shRNA and treated with 1 nM calyculinA (CalyA) for 3 h before fixation. Mean ± SEM are represented in the adjacent graph. Statistical differences in **(C,D)** were analyzed by a Kruskal-Wallis test followed by a Dunn’s multiple comparisons post-test. *p < 0.05, **p < 0.01, ***p < 0.001.

How can actin cytoskeleton instability resulting from lack of P2Y1 activity modify ankyrinG in the AIS? Recently published work has highlighted the role of myosin II and myosin light chain phosphorylation (pMLC) on AIS (Evans et al., 2017; Berger et al., 2018). In platelets, ADP and P2Y1 activity contribute to shape changes by increasing pMLC levels in a calcium-calmodulin dependent manner (Paul et al., 1999). Therefore, we analyzed pMLC levels and localization in 7 DIV shP2Y1 neurons (Figure 8A). As previously demonstrated (Berger et al., 2018), pMLC was localized at the AIS in 7 DIV control neurons. However, pMLC staining in the AIS was 20% lower in shP2Y1 neurons (Figure 8B). Next, we analyzed whether treating neurons with CalyculinA to inhibit PP1/PP2A, the phosphatases acting on pMLC, could recover pMLC levels and, consequently, ankyrinG. Calyculin A (1 nM) treatment for 3 h increased pMLC intensity at the AIS of control neurons by 40%, while in non-treated shP2Y1 neurons it was reduced by 31% (Figure 8C). Calyculin A treatment in shP2Y1 neurons increased pMLC intensity by 25% when compared to control neurons without Calyculin A treatment. AnkyrinG intensity analysis in the same experimental conditions revealed a recovery of ankyrinG in shP2Y1 neurons treated with Calyculin A (87.82 ± 4.01%) compared to shP2Y1 neurons (72.04 ± 3.08%), which did not completely reach reference control levels (100 ± 4.16%, Figure 8D). Interestingly, 3 h treatment of calyculin A in control neurons reduced ankyrinG intensity (78.5 ± 3.89%). This may suggest that increased pMLC levels are also detrimental to ankyrinG clustering at the AIS, as happened with F-actin stabilization with jasplakinolide (Figure 7F).

Altogether, our results underscore the role of P2Y1 as one of the receptors that contribute to AIS plasticity during early events of AIS maturation. Further investigations are required to completely understand the signaling pathway and interactions with other receptors and neurotransmitters.

## Discussion

Relevant studies have recently underscored the importance of AIS in neurodevelopmental disorders and neurodegenerative diseases (Kaphzan et al., 2011; Sun et al., 2014; van der Werf et al., 2017). Changes in AIS protein composition have been detected in models of disease, such as Angelman syndrome or Alzheimer’s disease (Kaphzan et al., 2011; Sun et al., 2014). However, our understanding of AIS composition, development and regulation is still limited. Which are the extrinsic and intrinsic mechanisms that modulate the AIS? The AIS has been proven to respond to external stimuli with a high degree of structural plasticity. However, our knowledge of the receptors, neurotransmitters and extracellular factors that modulate AIS remains limited. Understanding how the AIS can be deregulated may contribute to control and recover physiological neuronal function.

Prior studies demonstrated that the purinergic receptor P2X7 modulates AIS composition under pathological conditions (Del Puerto et al., 2015). Due to its relation with the P2Y1 receptor, the objective of our study was to decipher whether the purinergic receptor P2Y1 plays a role in AIS development and maintenance. Our results show that ADP-P2Y1 receptor contributes to the initial accumulation of ankyrinG at the AIS. P2Y1 suppression or inhibition decreases ankyrinG levels, as well as, voltage-gated sodium channels, βIV-spectrin and pMLC. P2Y1 is necessary for a proper elongation of the axon in early stages and is expressed in dendrites and axon terminals in cultured hippocampal neurons (del Puerto et al., 2012). P2Y1 expression is not detected in AIS, as previously mentioned for P2X7 (Del Puerto et al., 2015). Previous studies demonstrated a correlation between dendrites length or caliber and AIS properties (Hamada et al., 2016; Tapia et al., 2017). Our results show that, in control neurons, dendrites length and ankyrinG density is correlated, albeit there is no statistical correlation between both parameters after P2Y1 suppression. This suggests that P2Y1-mediated ankyrinG density regulation entails additional mechanisms triggered by P2Y1 alone or in coordination with other receptors. In fact, ankyrinG levels can be recovered through ATP-P2X7 receptor inhibition in neurons lacking P2Y1 receptor. A previous study demonstrated that high extracellular concentrations of ATP decrease ankyrinG density through P2X7 and calpain activation in mature cultured hippocampal neurons (Del Puerto et al., 2015). Here, we show that extracellular ADP or P2Y1 inhibition do not modify ankyrinG density in these mature neurons. However, through P2Y1 receptor, ADP is necessary to maintain ankyrinG density in young cultured hippocampal neurons. The P2Y1 regulation coincides with the initial stages of AIS development, between 4 and 10 DIV, when the diffusion barrier is being generated (Brachet et al., 2010) and is lost once the AIS achieve a certain degree of maturity and stability, around 10–14 DIV. In fact, P2Y1 suppression or ADP treatment after 7 DIV has no effect on ankyrinG density, while P2Y1 inactivation at 7 DIV or before decreases ankyrinG density. As happened during early axonal elongation (del Puerto et al., 2012), BBG-mediated P2X7 inhibition reverts the effects of P2Y1 suppression on ankyrinG density. However, P2X7 inhibition mediated recovery was not as high as previously observed in mature neurons (Del Puerto et al., 2015), suggesting that P2Y1 receptors decrease their expression or function during AIS development, while the importance of P2X7 increases. This recovery can be the result of decreased P2X7-mediated calcium entry and reduced calpain activation, but other mechanisms may also participate in this regulation. P2Y1 and P2X7 receptors are important regulators of neuronal excitability. P2Y1 receptors are expressed postsynaptically on dendrites of pyramidal cells and presynaptic terminals and participate in the regulation of neuronal plasticity (Barańska et al., 2017). Interestingly, P2X7 and P2Y1 are balanced over the regulation of cAMP and PKA activity, where P2Y1 contributes to increased cAMP levels (del Puerto et al., 2012). It was postulated that a PKA and phosphatases PP1 and calcineurin loop may regulate sodium channels phosphorylation modulating spike firing that correlates with AIS length in dentate granule cells (DGCs). In this study (Evans et al., 2015), PKA activation did not modify AIS length but did reverse the depolarization-induced changes in sodium channels properties, increasing their phosphorylation and decreasing conductance. Our results show P2Y1 inhibition or suppression does not alter AIS length, but rather proximally shifts the maximum ankyrinG intensity while decreasing ankyrinG density. It remains to be determined whether this shift is due to PKA activity levels or modifications of sodium channels conductance. Nevertheless, our results show that PP1/PP2A inhibition with calyculin A is able to counteract the lack of P2Y1 expression, which may balance PKA-phosphatases loop. On the other hand, P2Y1 may participate in AIS modulation through regulation of its actin cytoskeleton. In support, P2Y1 regulates Rac and actin cytoskeleton in other cell types (Soulet et al., 2005). We show that F-actin distribution and intensity in AIS is modified after P2Y1 suppression, and actin cytoskeleton stabilization with jasplakinolide recovers ankyrinG density. In fact, short-time treatment with potassium chloride decreases ankyrinG density at the AIS while at the same time actin is destabilized (Berger et al., 2018). This may be caused by decreased phosphorylation of myosin II light chain ( pMLC) at the AIS (Berger et al., 2018). Our results demonstrate that P2Y1 suppression decreases pMLC levels at the AIS at the same time that ankyrinG density is reduced in a similar percentage. The lack of P2Y1 receptor is compensated by inhibition of MLC phosphatase using calyculin A. Interestingly, P2Y1 agonist, 2MeSADP, increases pMLC phosphorylation in platelets generating a shape change (Getz et al., 2010), through a Gq dependent mechanism. All these data suggest that P2Y1 receptor participate in the global regulation of AIS cytoskeleton dynamics during its initial development.

## Conclusion

In conclusion, our work demonstrates that P2Y1 receptor participates only during initial AIS development. This may represent decreased expression or function during neuronal maturation, while other receptors, such as P2X7, increase their participation and function in more mature neurons. P2Y1 expression levels or functional activation/deactivation in coordination with other receptors and mechanisms may participate in the fine modulation of AIS development. Future work will be necessary to completely understand the molecular mechanisms underlying P2Y1 receptor; both at the level of plasticity and the modulation of AIS cytoskeleton.

## Data Availability

All datasets generated for this study are included in the manuscript.

## Ethics Statement

Animals were housed in a room at controlled temperature and relative humidity with alternating 12 h light and dark cycles and free access to food and water “*ad libitum*.” Animal care protocols used in our laboratory are in conformity with the appropriate national legislation (53/2013, BOE no. 1337) and guidelines of the Council of the European Communities (2010/63/UE). All protocols were previously approved by the CSIC bioethics committee.

## Author Contributions

JG, WZ and AB conceived and designed the experiments. JG, WZ, AB, MC and MB performed the experiments and data acquisition. JG and WZ analyzed and interpreted the data. JG wrote the manuscript. AB, MB and MC contributed to acquiring and analyzing data. All authors read and approved the final manuscript.

## Conflict of Interest Statement

The authors declare that the research was conducted in the absence of any commercial or financial relationships that could be construed as a potential conflict of interest.
